# Benzene-1,2-di(aminium) naphthalene-1,5-disulfonate methanol monosolvate trihydrate

**DOI:** 10.1107/S1600536812018284

**Published:** 2012-04-28

**Authors:** Shan Gao, Seik Weng Ng

**Affiliations:** a Key Laboratory of Functional Inorganic Material Chemistry, Ministry of Education, Heilongjiang University, Harbin 150080, People’s Republic of China; b Department of Chemistry, University of Malaya, 50603 Kuala Lumpur, Malaysia; c Chemistry Department, Faculty of Science, King Abdulaziz University, PO Box 80203 Jeddah, Saudi Arabia

## Abstract

In the title salt, C_6_H_10_N_2_^2+^·C_10_H_6_O_6_S_2_^2−^·CH_3_OH·3H_2_O, the cation lies on a mirror plane and the anion on a center of inversion. One lattice water mol­ecule is located on a mirror plane, another is equally disordered over two sites. The methanol solvent mol­ecule is disordered about a mirror plane. In the crystal, the cations, anions, water and methanol mol­ecules are linked by O—H⋯O and N—H⋯O hydrogen bonds, forming a three-dimensional network.

## Related literature

For other diammonium napthalene-1,5-disulfonates, see: Wei (2011 ▶); Zhu *et al.* (2009 ▶).
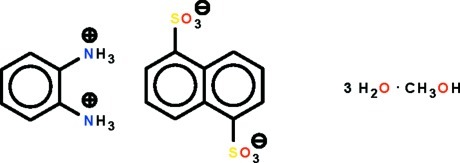


## Experimental

### 

#### Crystal data

C_6_H_10_N_2_^2+^·C_10_H_6_O_6_S_2_^2−^·CH_4_O·3H_2_O
*M* *_r_* = 482.52
Monoclinic, 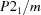

*a* = 8.1727 (15) Å
*b* = 13.681 (2) Å
*c* = 9.5173 (15) Å
β = 98.390 (5)°
*V* = 1052.8 (3) Å^3^
*Z* = 2
Mo *K*α radiation
μ = 0.31 mm^−1^
*T* = 293 K
0.25 × 0.22 × 0.19 mm

#### Data collection

Rigaku R-AXIS RAPID IP diffractometer
Absorption correction: multi-scan (*ABSCOR*; Higashi, 1995 ▶) *T*_min_ = 0.926, *T*_max_ = 0.943
10378 measured reflections
2503 independent reflections
1935 reflections with *I* > 2σ(*I*)
*R*_int_ = 0.044

#### Refinement

*R*[*F* ^2^ > 2σ(*F* ^2^)] = 0.055
*wR*(*F* ^2^) = 0.176
*S* = 1.13
2503 reflections
174 parameters
H-atom parameters constrained
Δρ_max_ = 0.85 e Å^−3^
Δρ_min_ = −0.41 e Å^−3^

### 

Data collection: *RAPID-AUTO* (Rigaku, 1998 ▶); cell refinement: *RAPID-AUTO*; data reduction: *CrystalClear* (Rigaku/MSC, 2002 ▶); program(s) used to solve structure: *SHELXS97* (Sheldrick, 2008 ▶); program(s) used to refine structure: *SHELXL97* (Sheldrick, 2008 ▶); molecular graphics: *X-SEED* (Barbour, 2001 ▶); software used to prepare material for publication: *publCIF* (Westrip, 2010 ▶).

## Supplementary Material

Crystal structure: contains datablock(s) global, I. DOI: 10.1107/S1600536812018284/xu5520sup1.cif

Structure factors: contains datablock(s) I. DOI: 10.1107/S1600536812018284/xu5520Isup2.hkl

Supplementary material file. DOI: 10.1107/S1600536812018284/xu5520Isup3.cml

Additional supplementary materials:  crystallographic information; 3D view; checkCIF report

## Figures and Tables

**Table 1:** Hydrogen-bond geometry (Å, °)

*D*—H⋯*A*	*D*—H	H⋯*A*	*D*⋯*A*	*D*—H⋯*A*
O4—H4⋯O3	0.84	1.94	2.783 (6)	175
O1*w*—H1*w*⋯O2*w*	0.84	2.32	2.915 (6)	128
O1*w*—H1*w*⋯O2*w*′	0.84	2.19	2.799 (7)	130
O2*w*—H2*w*2⋯O3^i^	0.84	2.35	2.873 (5)	121
O2*w*′—H2*w*4⋯O3^i^	0.84	2.25	2.808 (6)	124
N1—H12⋯O1^ii^	0.88	2.07	2.880 (3)	153
N1—H11⋯O2*w*′	0.88	2.03	2.816 (5)	149
N2—H22⋯O1	0.88	1.99	2.815 (3)	156
N2—H21⋯O1*w*^iii^	0.88	1.88	2.735 (4)	164

Symmetry codes: (i) 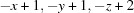; (ii) 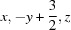; (iii) 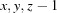.

**Fig. 1.**
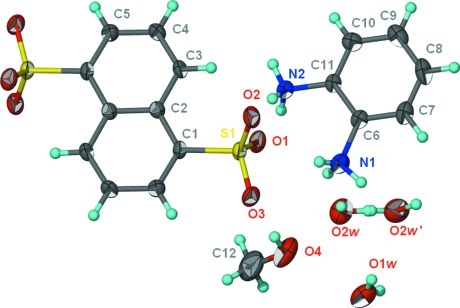
Thermal ellipsoid plot (Barbour, 2001) of C6H10N22+.C10H6O6S22–.3H2O.CH3OH at the 50% probability level; hydrogen atoms are drawn as spheres of arbitrary radius. The symmetry-related atoms of the anion are not labeled.

## supplementary crystallographic information

### Comment

Naphthalene-1,5-disulfonic acid has been characterized as several diammonium salts, *e.g.*, as the ethane-1,2-diammonium (Zhu *et al.*, 2009), the piperizine-1,4-diium (Wei, 2011) derivatives. In the salt, C_6_H_10_N_2_^2+.^C_10_H_6_O_6_S_2_^2–.^3H_2_O^.^CH_3_OH (Scheme I, Fig. 1), the cation lies on a mirror plane and the anion on a center-of-inversion. The cation, anion, water and methanol molecules are linked by O–H···O hydrogen bonds to form a three-dimensional network (Table 1).

### Experimental

A methanol solution (5 ml) of 1,2-benzenediamine (1 mmol, 108 mg) was added to an aqueous solution (5 ml) of 1,5-naphthalenedisulfonic acid tetrahydrate (0.5 mmol, 180 mg). The mixture was heated at 343 K until the reactants dissolved completely. The solution was filtered; colorless crystals were isolated after several days.suitable for X-ray diffraction were isolated from the filtrate afterfour days.

### Refinement

Carbon- and nitrogen-bound H-atoms were placed in calculated positions (C–H 0.93, N–H 0.88 Å) and were included in the refinement in the riding model approximation, with *U*(H) set to 1.2*U*(C,*N*). The water H-atoms were placed in chemcally sensible positions on the basis of hydrogen bonding interactions (O–H 0.84 Å) and their temperature factors similar tied. One of the water molecules is disordered over a center-of-inversion.

### Crystal data

**Table:**

C_6_H_10_N_2_^2+^·C_10_H_6_O_6_S_2_^2^^−^·CH_4_O·3H_2_O	*F*(000) = 508
*M**_r_* = 482.52	*D*_x_ = 1.522 Mg m^−^^3^
Monoclinic, *P*2_1_/*m*	Mo *K*α radiation, λ = 0.71073 Å
Hall symbol: -P 2yb	Cell parameters from 6427 reflections
*a* = 8.1727 (15) Å	θ = 3.1–27.5°
*b* = 13.681 (2) Å	µ = 0.31 mm^−^^1^
*c* = 9.5173 (15) Å	*T* = 293 K
β = 98.390 (5)°	Prism, colorless
*V* = 1052.8 (3) Å^3^	0.25 × 0.22 × 0.19 mm
*Z* = 2	

### Data collection

**Table:**

Rigaku R-AXIS RAPID IP diffractometer	2503 independent reflections
Radiation source: fine-focus sealed tube	1935 reflections with *I* > 2σ(*I*)
Graphite monochromator	*R*_int_ = 0.044
ω scan	θ_max_ = 27.5°, θ_min_ = 3.1°
Absorption correction: multi-scan (*ABSCOR*; Higashi, 1995)	*h* = −10→10
*T*_min_ = 0.926, *T*_max_ = 0.943	*k* = −16→17
10378 measured reflections	*l* = −12→12

### Refinement

**Table:**

Refinement on *F*^2^	Primary atom site location: structure-invariant direct methods
Least-squares matrix: full	Secondary atom site location: difference Fourier map
*R*[*F*^2^ > 2σ(*F*^2^)] = 0.055	Hydrogen site location: inferred from neighbouring sites
*wR*(*F*^2^) = 0.176	H-atom parameters constrained
*S* = 1.13	*w* = 1/[σ^2^(*F*_o_^2^) + (0.0909*P*)^2^ + 0.6772*P*] where *P* = (*F*_o_^2^ + 2*F*_c_^2^)/3
2503 reflections	(Δ/σ)_max_ = 0.001
174 parameters	Δρ_max_ = 0.85 e Å^−^^3^
0 restraints	Δρ_min_ = −0.41 e Å^−^^3^

### Fractional atomic coordinates and isotropic or equivalent isotropic displacement parameters (Å^2^)

**Table:**

	*x*	*y*	*z*	*U*_iso_*/*U*_eq_	Occ. (<1)
S1	0.71163 (7)	0.51770 (5)	0.72850 (6)	0.0322 (2)	
O1	0.6469 (2)	0.61258 (14)	0.6776 (2)	0.0425 (5)	
O2	0.6097 (2)	0.43755 (15)	0.6701 (2)	0.0443 (5)	
O3	0.7450 (3)	0.51585 (15)	0.8836 (2)	0.0433 (5)	
O1w	0.5509 (5)	0.7500	1.2108 (4)	0.0707 (10)	
H1w	0.4934	0.6998	1.1902	0.106*	
N1	0.4198 (5)	0.7500	0.7729 (4)	0.0509 (9)	
H11	0.3788	0.7500	0.8535	0.076*	
H12	0.4809	0.8025	0.7685	0.076*	
N2	0.4862 (4)	0.7500	0.4847 (3)	0.0368 (7)	
H21	0.4867	0.7500	0.3923	0.055*	
H22	0.5374	0.6975	0.5221	0.055*	
C1	0.9077 (3)	0.50513 (18)	0.6689 (3)	0.0300 (5)	
C2	0.9198 (3)	0.50489 (17)	0.5204 (3)	0.0292 (5)	
C3	0.7803 (3)	0.51357 (19)	0.4131 (3)	0.0338 (6)	
H3	0.6752	0.5191	0.4388	0.041*	
C4	0.7984 (3)	0.5140 (2)	0.2731 (3)	0.0387 (6)	
H4A	0.7057	0.5207	0.2045	0.046*	
C5	0.9552 (3)	0.5044 (2)	0.2309 (3)	0.0373 (6)	
H5	0.9659	0.5044	0.1349	0.045*	
C6	0.2850 (5)	0.7500	0.6545 (4)	0.0354 (8)	
C7	0.1240 (5)	0.7500	0.6818 (5)	0.0453 (10)	
H7	0.1025	0.7500	0.7751	0.054*	
C8	−0.0050 (5)	0.7500	0.5712 (6)	0.0527 (11)	
H8	−0.1133	0.7500	0.5904	0.063*	
C9	0.0245 (6)	0.7500	0.4325 (5)	0.0499 (10)	
H9	−0.0631	0.7500	0.3582	0.060*	
C10	0.1866 (5)	0.7500	0.4045 (4)	0.0431 (9)	
H10	0.2077	0.7500	0.3111	0.052*	
C11	0.3153 (4)	0.7500	0.5139 (4)	0.0315 (7)	
O4	0.7361 (8)	0.6961 (4)	1.0177 (6)	0.0782 (17)	0.50
H4	0.7426	0.6406	0.9812	0.117*	0.50
C12	0.8778 (11)	0.751 (2)	1.0006 (8)	0.090 (3)	0.5
H12A	0.8778	0.8110	1.0533	0.135*	0.50
H12B	0.8767	0.7658	0.9018	0.135*	0.50
H12C	0.9753	0.7143	1.0351	0.135*	0.50
O2w	0.4770 (6)	0.6054 (4)	0.9855 (5)	0.0635 (13)	0.50
H2w1	0.3897	0.6342	0.9987	0.095*	0.50
H2w2	0.4637	0.5451	0.9955	0.095*	0.50
O2w'	0.2979 (7)	0.6729 (4)	1.0117 (5)	0.0657 (13)	0.50
H2w3	0.3789	0.6375	1.0001	0.099*	0.50
H2w4	0.2196	0.6366	1.0271	0.099*	0.50

### Atomic displacement parameters (Å^2^)

**Table:**

	*U*^11^	*U*^22^	*U*^33^	*U*^12^	*U*^13^	*U*^23^
S1	0.0272 (4)	0.0378 (4)	0.0335 (4)	0.0033 (2)	0.0108 (2)	0.0024 (2)
O1	0.0421 (11)	0.0428 (11)	0.0451 (10)	0.0138 (9)	0.0152 (8)	0.0056 (9)
O2	0.0313 (10)	0.0483 (12)	0.0553 (12)	−0.0061 (8)	0.0127 (8)	−0.0051 (10)
O3	0.0434 (11)	0.0557 (12)	0.0333 (10)	0.0051 (9)	0.0143 (8)	0.0054 (9)
O1w	0.086 (3)	0.083 (3)	0.0472 (18)	0.000	0.0224 (18)	0.000
N1	0.044 (2)	0.074 (3)	0.0366 (17)	0.000	0.0125 (15)	0.000
N2	0.0354 (17)	0.0422 (17)	0.0341 (16)	0.000	0.0096 (13)	0.000
C1	0.0241 (12)	0.0339 (12)	0.0335 (13)	0.0028 (9)	0.0096 (9)	0.0008 (10)
C2	0.0265 (13)	0.0297 (12)	0.0314 (12)	0.0016 (9)	0.0045 (9)	0.0021 (10)
C3	0.0219 (12)	0.0428 (14)	0.0367 (13)	0.0009 (10)	0.0050 (10)	0.0003 (11)
C4	0.0281 (13)	0.0520 (17)	0.0343 (14)	0.0034 (11)	−0.0007 (10)	0.0015 (11)
C5	0.0339 (14)	0.0480 (15)	0.0299 (12)	0.0026 (11)	0.0045 (10)	0.0024 (11)
C6	0.038 (2)	0.0342 (18)	0.0360 (18)	0.000	0.0101 (15)	0.000
C7	0.043 (2)	0.044 (2)	0.054 (2)	0.000	0.0200 (19)	0.000
C8	0.032 (2)	0.048 (2)	0.081 (3)	0.000	0.016 (2)	0.000
C9	0.038 (2)	0.045 (2)	0.064 (3)	0.000	−0.0007 (19)	0.000
C10	0.043 (2)	0.045 (2)	0.040 (2)	0.000	0.0021 (17)	0.000
C11	0.0317 (18)	0.0271 (16)	0.0372 (18)	0.000	0.0100 (14)	0.000
O4	0.098 (4)	0.069 (3)	0.079 (4)	−0.002 (3)	0.050 (3)	−0.012 (3)
C12	0.097 (6)	0.093 (6)	0.086 (5)	0.035 (14)	0.031 (4)	−0.009 (16)
O2w	0.067 (3)	0.060 (3)	0.066 (3)	0.004 (2)	0.020 (2)	0.002 (2)
O2w'	0.071 (3)	0.077 (3)	0.051 (3)	−0.010 (3)	0.015 (2)	0.011 (2)

### Geometric parameters (Å, º)

**Table:**

S1—O2	1.439 (2)	C6—C7	1.377 (5)
S1—O1	1.4578 (19)	C6—C11	1.396 (5)
S1—O3	1.461 (2)	C7—C8	1.377 (7)
S1—C1	1.785 (2)	C7—H7	0.9300
O1w—H1w	0.8391	C8—C9	1.376 (7)
N1—C6	1.456 (5)	C8—H8	0.9300
N1—H11	0.8800	C9—C10	1.389 (6)
N1—H12	0.8800	C9—H9	0.9300
N2—C11	1.463 (4)	C10—C11	1.368 (5)
N2—H21	0.8800	C10—H10	0.9300
N2—H22	0.8800	O4—C12	1.41 (2)
C1—C5^i^	1.367 (4)	O4—H4	0.8400
C1—C2	1.430 (3)	C12—H12A	0.9600
C2—C3	1.420 (4)	C12—H12B	0.9600
C2—C2^i^	1.428 (5)	C12—H12C	0.9600
C3—C4	1.362 (4)	O2w—H2w1	0.8401
C3—H3	0.9300	O2w—H2w2	0.8400
C4—C5	1.404 (4)	O2w—H2w3	0.9423
C4—H4A	0.9300	O2w'—H2w1	0.9412
C5—C1^i^	1.367 (4)	O2w'—H2w3	0.8400
C5—H5	0.9300	O2w'—H2w4	0.8400
			
O2—S1—O1	112.86 (13)	C1^i^—C5—H5	120.1
O2—S1—O3	112.92 (12)	C4—C5—H5	120.1
O1—S1—O3	110.88 (12)	C7—C6—C11	119.2 (4)
O2—S1—C1	107.45 (12)	C7—C6—N1	119.3 (3)
O1—S1—C1	106.22 (12)	C11—C6—N1	121.5 (3)
O3—S1—C1	105.98 (12)	C8—C7—C6	120.1 (4)
C6—N1—H11	109.5	C8—C7—H7	119.9
C6—N1—H12	109.5	C6—C7—H7	119.9
H11—N1—H12	109.5	C9—C8—C7	120.8 (4)
C11—N2—H21	109.5	C9—C8—H8	119.6
C11—N2—H22	109.5	C7—C8—H8	119.6
H21—N2—H22	109.5	C8—C9—C10	119.3 (4)
C5^i^—C1—C2	121.4 (2)	C8—C9—H9	120.3
C5^i^—C1—S1	118.00 (19)	C10—C9—H9	120.3
C2—C1—S1	120.56 (19)	C11—C10—C9	120.2 (4)
C3—C2—C2^i^	119.0 (3)	C11—C10—H10	119.9
C3—C2—C1	123.1 (2)	C9—C10—H10	119.9
C2^i^—C2—C1	117.9 (3)	C10—C11—C6	120.4 (3)
C4—C3—C2	120.9 (2)	C10—C11—N2	120.3 (3)
C4—C3—H3	119.6	C6—C11—N2	119.3 (3)
C2—C3—H3	119.6	H2w1—O2w—H2w2	108.5
C3—C4—C5	120.9 (2)	H2w2—O2w—H2w3	108.3
C3—C4—H4A	119.5	H2w1—O2w'—H2w4	109.5
C5—C4—H4A	119.5	H2w3—O2w'—H2w4	108.5
C1^i^—C5—C4	119.9 (2)		
			
O2—S1—C1—C5^i^	−120.4 (2)	C3—C4—C5—C1^i^	−0.3 (4)
O1—S1—C1—C5^i^	118.6 (2)	C11—C6—C7—C8	0.000 (2)
O3—S1—C1—C5^i^	0.6 (3)	N1—C6—C7—C8	180.000 (1)
O2—S1—C1—C2	59.5 (2)	C6—C7—C8—C9	0.000 (2)
O1—S1—C1—C2	−61.5 (2)	C7—C8—C9—C10	0.000 (2)
O3—S1—C1—C2	−179.51 (19)	C8—C9—C10—C11	0.000 (1)
C5^i^—C1—C2—C3	179.3 (3)	C9—C10—C11—C6	0.0
S1—C1—C2—C3	−0.5 (3)	C9—C10—C11—N2	180.0
C5^i^—C1—C2—C2^i^	−0.5 (4)	C7—C6—C11—C10	0.000 (1)
S1—C1—C2—C2^i^	179.7 (2)	N1—C6—C11—C10	180.000 (1)
C2^i^—C2—C3—C4	−0.9 (4)	C7—C6—C11—N2	180.000 (1)
C1—C2—C3—C4	179.3 (2)	N1—C6—C11—N2	0.000 (1)
C2—C3—C4—C5	0.9 (4)		

Symmetry code: (i) −*x*+2, −*y*+1, −*z*+1.

### Hydrogen-bond geometry (Å, º)

**Table:**

*D*—H···*A*	*D*—H	H···*A*	*D*···*A*	*D*—H···*A*
O4—H4···O3	0.84	1.94	2.783 (6)	175
O1*w*—H1*w*···O2*w*	0.84	2.32	2.915 (6)	128
O1*w*—H1*w*···O2*w*′	0.84	2.19	2.799 (7)	130
O2*w*—H2*w*2···O3^ii^	0.84	2.35	2.873 (5)	121
O2*w*′—H2*w*4···O3^ii^	0.84	2.25	2.808 (6)	124
N1—H12···O1^iii^	0.88	2.07	2.880 (3)	153
N1—H11···O2*w*′	0.88	2.03	2.816 (5)	149
N2—H22···O1	0.88	1.99	2.815 (3)	156
N2—H21···O1*w*^iv^	0.88	1.88	2.735 (4)	164

Symmetry codes: (ii) −*x*+1, −*y*+1, −*z*+2; (iii) *x*, −*y*+3/2, *z*; (iv) *x*, *y*, *z*−1.

## References

[bb1] Barbour, L. J. (2001). *J. Supramol. Chem.* **1**, 189–191.

[bb2] Higashi, T. (1995). *ABSCOR* Rigaku Corporation, Tokyo, Japan.

[bb3] Rigaku (1998). *RAPID-AUTO* Rigaku Corporation, Tokyo, Japan.

[bb4] Rigaku/MSC (2002). *CrystalClear* Rigaku/MSC Inc., The Woodlands, Texas, USA.

[bb5] Sheldrick, G. M. (2008). *Acta Cryst.* A**64**, 112–122. 10.1107/S0108767307043930 18156677

[bb6] Wei, B. (2011). *Acta Cryst.* E**67**, o2811. 10.1107/S1600536811038955 PMC3201365 22065696

[bb7] Westrip, S. P. (2010). *J. Appl. Cryst.* **43**, 920–925.

[bb8] Zhu, Z.-B., Gao, S. & Ng, S. W. (2009). *Acta Cryst.* E**65**, o2658. 10.1107/S1600536809039762 PMC2971418 21578270

